# Influence of alcohol sensitivity on bone metastases and skeletal-related events in primary operable breast cancer: A retrospective cohort study

**DOI:** 10.1371/journal.pone.0269335

**Published:** 2022-06-03

**Authors:** Naoko Tanda, Hiroshi Tada, Jumpei Washio, Nobuhiro Takahashi, Takanori Ishida, Takeyoshi Koseki

**Affiliations:** 1 Division of Preventive Dentistry, Graduate School of Dentistry, Tohoku University, Sendai, Japan; 2 Department of Breast and Endocrine Surgical Oncology, Graduate School of Medicine, Tohoku University, Sendai, Japan; 3 Division of Oral Ecology and Biochemistry, Graduate School of Dentistry, Tohoku University, Sendai, Japan; Universita degli Studi della Campania Luigi Vanvitelli, ITALY

## Abstract

**Background:**

Bone metastases in breast cancer patients are a common concern for medical doctors and dentists. Bone-modifying agents, which are necessary to prevent skeletal-related events (SREs), are associated with osteonecrosis of the jaw as an adverse side effect. Hypersensitivity to alcohol is an unfavorable response caused by deficiency of aldehyde dehydrogenase-2 (ALDH2) activity. Inactive ALDH2 is associated with osteoporosis, but its influence on bone metastases is unclear. The aim of our study was to evaluate the effects of alcohol sensitivity on bone metastases and SREs in primary operable breast cancer patients.

**Methods:**

We retrospectively analyzed patients who were administered docetaxel, an anti-tumor agent, for histologically diagnosed breast cancer between April 2004 and September 2015. Alcohol sensitivity was assessed based on medical records of hypersensitivity to alcohol. The primary endpoint was time to bone metastases and the secondary endpoint was time to first SRE from the initial docetaxel administration. Data were stratified by alcohol sensitivity and tumor stages, and differences were estimated by the Kaplan-Meier method. Prognostic risk factors were analyzed by the multivariate Cox proportional hazards model.

**Results:**

The median follow-up period of patients with high sensitivity to alcohol (n = 45) was 54 months and that for those with low sensitivity (n = 287) was 64 months. Stratification by alcohol sensitivity revealed that tumor stage exhibited significant correlations with the cumulative incidence of bone metastases in low-sensitivity patients; however, no differences were found in high-sensitivity patients. In multivariate analysis, alcohol sensitivity was a significant prognostic risk factor for bone metastases (HR 2.721, 95% CI 1.268–5.841, *P* = 0.010).

**Conclusion:**

Alcohol sensitivity may be a prognostic risk factor for bone metastases. More detailed genetic investigations and metabolic analyses are needed.

## Introduction

Breast cancer is the most commonly diagnosed cancer among females and the leading cause of cancer death [1]. Bone is the most frequent metastatic site of breast cancer, and metastases cause a poor quality of life due to bone pain, pathological fracture, hypercalcemia, and spinal cord compression [2, 3]. Bone-modifying agents, such as bisphosphonates and denosumab, are clinically effective in delaying the onset of skeletal-related events (SREs) and reduce their incidence; however, both drugs are associated with medication-related osteonecrosis of the jaw (MRONJ) [2, 4, 5]. MRONJ is defined as exposed bone or bone that can be probed through an intraoral or extra oral fistula(e) in the maxillofacial region that does not heal within 8 weeks developing in a patient who received a bone-modifying agent or an angiogenic inhibitor agent with no history of head and neck radiation [6, 7]. Although MRONJ causes pain, malodor, and difficulty eating, and reduces the quality of life, it can be challenging to treat [8]. Preventive oral care methods, including comprehensive dental assessments and avoidance of modifiable risk factors, combined with effective oral health practices are recommended [6, 9]. MRONJ has become a common concern for dentists and medical doctors who are involved in the management of patients with a high risk of bone metastasis, and requires a multi-professional approach [6, 8, 10, 11].

Breast cancer cells in the bone microenvironment release soluble factors that engage osteoclasts and/or osteoblasts, resulting in bone breakdown. The breakdown of the bone matrix promotes the proliferation of cancer cells, creating a vicious cycle [12]. Breast cancer with bone metastasis is predominantly osteolytic. The outcome mainly depends on the impact of cancer cells on osteoblasts regardless of the role of osteoclasts in this process. Metastatic breast cancer cells or their conditioned media increase osteoblast apoptosis, and suppress osteoblast differentiation and expression of proteins required for new bone matrix formation [13]. Several studies suggested that aldehyde stress resulting from aldehyde dehydrogenase 2 (ALDH2) polymorphism leads to impaired osteoblastogenesis due to the lack of enzyme protection against aldehyde toxicity for osteoblasts and their progenitor cells [14–17].

Alcohol sensitivity has been studied as one of possible predictors of inactive ALDH2, as ALDH2 dysfunction contributes to various human diseases including esophageal and upper aerodigestive track cancers [17, 18]. Those with the dominant-negative form of ALDH2 protein (ALDH2*2) have high sensitivity to alcohol, which is associated with unpleasant responses, such as facial flushing, nausea, palpitations, and tachycardia, mainly in East Asian populations [18–21]. Yokoyama *et al*. assumed that individuals with current or former flushing had inactive ALDH2, and found that their responses to a simple flushing questionnaire yielded a sensitivity and specificity of approximately 90% for identifying ALDH2-deficient individuals among the Japanese general population of men and women aged 40 years or older [22–24]. Recently alcohol sensitivity has been reported to be a factor in the side effects of anticancer drugs such as paclitaxel or docetaxel containing alcohol as a solvent [25, 26]. The safety of paclitaxel for alcohol intolerance has been studied and patients suspected of alcohol intolerance by a screening question were found to carry inactive genotypes of *ALDH2* GA (heterozygous ALDH2 *1/*2) or *ALDH2* AA (homozygous ALDH2 *2/*2) [26]. Such chemotherapeutic agents are essential for breast cancer treatment; thus, it is necessary to clarify alcohol sensitivity to protect patients from adverse reactions.

Aldehyde dehydrogenase-2 deficiency, which causes high sensitivity to alcohol, is associated with osteoporosis [27]; however, its influence on bone metastases is unclear. We hypothesized that high sensitivity to alcohol is a prognostic factor for bone metastases, and evaluated the effects of alcohol sensitivity on bone metastases and SREs in primary operable breast cancer patients.

## Materials and methods

All procedures were approved by the Institutional Review Board at Tohoku University Graduate School of Dentistry (No. 2018-3-20). Patients were not required to give informed consent to the study because the analysis used anonymous clinical data that were obtained after each patient agreed to treatment by written consent. The patients were given the opportunity to opt out of the study at any time, which was announced on the website of Tohoku University Graduate School of Dentistry (http://www.dent.tohoku.ac.jp/general/open/info_02/files/2018-20.pdf).

### Patients

Female patients administered docetaxel, an anti-cancer agent, for pathologically confirmed breast cancer between April 2004 and September 2015 at Tohoku University Hospital were included in this study. The data cutoff point was August 31, 2018. The medical records of each patient were retrospectively reviewed. Patients were regarded as eligible if they were aged 20 years or older, and had no recurrence or distant metastases (stage I, II, or III based on the UICC TNM classification) at the first administration of docetaxel. Docetaxel was prepared by dissolving the formulation in a solvent containing 13% ethanol. Alcohol-hypersensitive patients required ethanol-free preparations [25]. Patients with skin flushing or other uncomfortable reactions by alcohol according to their medical records and who required “alcohol-free” orders for docetaxel were assessed as having high sensitivity to alcohol. Alcohol-free orders that were requested because the patient was driving were excluded from the definition. Four hundred forty-six female patients with breast cancer who were administered docetaxel for cancer were extracted from medical records. Stage IV (n = 95) and recurrence (n = 19) were excluded from the study. Data from 332 patients were used for analysis. Forty-five patients had high sensitivity to alcohol, whereas 287 patients had low sensitivity to alcohol.

### Outcome

Collected data included alcohol sensitivity (high, low), age (50 and older, younger than 50 years), clinical stage at primary diagnosis (stage I, stages II and III), local immunohistochemistry evaluation for estrogen and progesterone receptor status (ER-negative, ER-positive, PgR-negative, PgR-positive), human epidermal growth factor receptor 2 (HER2) expression (HER2-negative, HER2-positive), bone metastasis, and SREs. Types of SREs, administration of bisphosphonates or denosumab and incidence of MRONJ, were also collected from records of patients who developed bone metastasis. Bone metastasis was diagnosed using imaging studies, physical examination, or symptoms. SREs were defined as pathological fractures, need for radiation or surgical interventions to bone, spinal cord compression, bone pain, and hypercalcemia [28]. The primary endpoint of the study was time to bone metastasis, defined as the interval from docetaxel administration to bone metastasis. The secondary endpoint was time to SREs, defined as the interval from docetaxel administration to the first SRE. The incidences of bone metastasis, pathological fractures, and development of MRONJ were also compared between patients with low sensitivity to alcohol and those with high sensitivity to alcohol.

### Statistical analyses

Categorical data were compared between groups using chi-square test or Fisher’s two-sided exact test as appropriate. Continuous data are presented as the median (interquartile range) and were compared by Mann-Whitney U test. Kaplan-Meier method with a log-rank test was used to estimate and compare the cumulative incidence of bone metastases and SREs. Data were stratified by alcohol sensitivity and tumor stages and a log-rank test was conducted in each stratum. Univariate analysis and multivariate analysis were performed using the Cox proportional hazards model to investigate associations between prognostic variables and time to bone metastasis or time to SREs.

Variables with *P* < 0.05 in univariate analysis were evaluated as potential covariates in multivariate analysis. A *P*-value less than 0.05 was considered significant. IBM SPSS Statistics for Macintosh (Version 25.0, IBM Corp., Armonk, NY) was used for statistical analysis.

## Results

### Baseline characteristics

**Fig 1:**
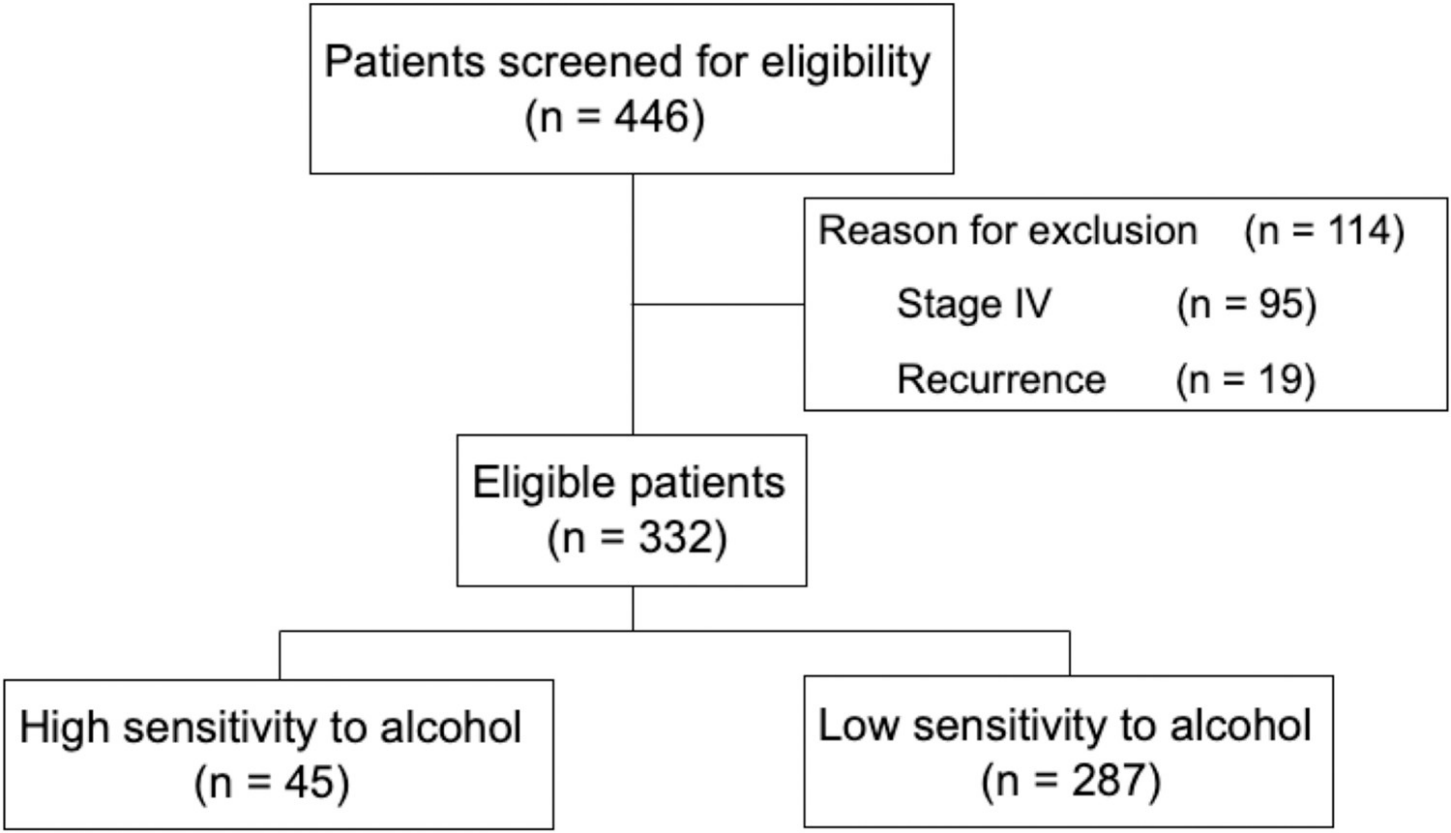
Flow diagram of the patients included in the analysis.

Four hundred forty-six female patients with breast cancer were extracted from electric medical records for use of the anti-cancer agent docetaxel. Stage IV (n = 95) and recurrence (n = 19) were excluded from the study. Data from 332 patients were available for analysis. Forty-five patients had high sensitivity to alcohol, whereas the others had low sensitivity to alcohol (Fig 1).

Clinicopathological characteristics by sensitivity to alcohol are shown in Table 1. The clinical characteristics of age, staging, ER, PgR, and HER2 were similar between the two groups. The median follow-up period was 54 months (interquartile rage 37–77 months) in patients with high sensitivity to alcohol and 64 months (interquartile rage 43–93 months) in patients with low sensitivity. The period was significantly shorter in patients with high sensitivity (*P* = 0.037). The median time to bone metastasis and time to first SRE were also significantly shorter in patients with high sensitivity to alcohol.

**Table 1:** Clinicopathological characteristics by sensitivity to alcohol.

	Low sensitivity (n = 287)	High sensitivity (n = 45)	*P-*value
Age (years)			0.100
< 50	107 (37.3%)	23 (51.1%)	
≥ 50	180 (62.7%)	22 (48.9%)	
Primary staging			0.558
I	59 (20.6%)	11 (24.4%)	
II and III	228 (79.4%)	34 (75.6%)	
ER status			0.857
negative	78 (27.2%)	11 (24.4%)	
positive	209 (72.8%)	34 (75.6%)	
PgR status			0.870
negative	110 (38.2%)	18 (40.0%)	
positive	177 (61.7%)	27 (60.0%)	
HER2 status			0.086
negative	226 (78.7%)	30 (66.7%)	
positive	61 (21.3%)	15 (33.3%)	
Time (months)			
to bone metastasis	63 (41–92)	50 (34–72)	0.016
to SREs	73 (43–92.5)	50 (36–72)	0.017
Follow-up period (months)	64 (43–93)	54 (37–77)	0.037

Data are n (%) or median (interquartile range, IQR). Differences in distributions of categorical and continuous variables between groups were examined using Fisher’s two-sided exact test and Mann-Whitney U test, respectively.

SREs, skeletal-related events; ER, estrogen receptor; PgR, progesterone receptor; HER2, human epidermal growth factor receptor 2

### Incidence of bone metastases and SREs stratified by alcohol sensitivity and tumor stages

Bone metastasis developed in 9 of 45 patients with high sensitivity to alcohol (20%) and in 26 of 287 patients with low sensitivity to alcohol (9.1%). SREs were observed in 4 of 45 patients with high sensitivity to alcohol (8.9%) and in 10 of 287 patients with low sensitivity to alcohol (3.5%). The incidence of bone metastases and SREs were stratified by alcohol sensitivity and tumor stages and compared between stages or sensitivities in each stratum (Tables 2–5).

**Table 2:** Incidence of bone metastases stratified by alcohol sensitivity and tumor stages.

Strata			Number (%)	
			BM +	BM -
Alcohol sensitivity	low	stage I	1 (1.7)	58 (98.3)
		stage II	8 (6.2)	121 (93.8)
		stage III	17 (17.2)	82 (82.8)
	high	stage I	1 (9.1)	10 (90.9)
		stage II	4 (18.2)	18 (81.8)
		stage III	4 (33.3)	8 (66.7)
Tumor stage	stage I	low sensitivity	1 (1.7)	58 (98.3)
		high sensitivity	1 (9.1)	10 (90.9)
	stage II	low sensitivity	8 (6.2)	121 (93.8)
		high sensitivity	4 (18.2)	18 (81.8)
	stage III	low sensitivity	17 (17.2)	82 (82.8)
		high sensitivity	4 (33.3)	8 (66.7)

BM, bone metastases.

**Table 3:** Differences of bone metastases between stages in patients stratified by alcohol sensitivity and between sensitivities in patients stratified by tumor stages.

Strata			*P-*value

Alcohol sensitivity	low	stage I vs. II	0.241
		stage I vs. III	0.003
		stage II vs. III	0.005
	high	stage I vs. II	0.55
		stage I vs. III	0.157
		stage II vs. III	0.249
Tumor stage	stage I	low vs. high	0.101
	stage II	low vs. high	0.017
	stage III	low vs. high	0.125

The cumulative incidence of bone metastases was significantly higher in high-sensitivity patients with tumor stage II. There were no significant differences between sensitivities in stage I and in stage III. The cumulative incidence of bone metastases was significantly higher in stage III compared to stage I or stage II in low-sensitivity patients; however, there were no significant differences between stages in high-sensitivity patients (see S1 Fig).

**Table 4:** Incidence of SREs stratified by alcohol sensitivity and tumor stages.

Strata			Number (%)	
			SRE +	SRE -
Alcohol sensitivity	low	stage I	0 (0)	59 (100)
		stage II	5 (3.9)	124 (96.1)
		stage III	5(5.1)	94 (94.9)
	high	stage I	1 (9.1)	10 (90.9)
		stage II	1 (4.5)	21 (95.5)
		stage III	2 (16.7)	10 (83.3)
Tumor stage	stage I	low sensitivity	0 (0)	59 (100)
		high sensitivity	1 (9.1)	10 (90.9)
	stage II	low sensitivity	5 (3.9)	124 (96.1)
		high sensitivity	1 (4.5)	21 (95.5)
	stage III	low sensitivity	5(5.1)	94 (94.9)
		high sensitivity	2 (16.7)	10 (83.3)

SREs, skeletal-related events.

**Table 5:** Differences of SREs between stages in patients stratified by alcohol sensitivity and between sensitivities in patients stratified by tumor stages.

Strata			*P-*value
Alcohol sensitivity	low	stage I vs. II	0.141
		stage I vs. III	0.076
		stage II vs. III	0.579
	high	stage I vs. II	0.617
		stage I vs. III	0.637
		stage II vs. III	0.323
Tumor stage	stage I	low vs. high	0.013
	stage II	low vs. high	0.682
	stage III	low vs. high	0.079

The cumulative incidence of SREs was significantly higher in high-sensitivity patients in tumor stage I; however, there were no significant differences between sensitivities in other stages and between stages (see S2 Fig).

### Risk factors

**Fig 2:**
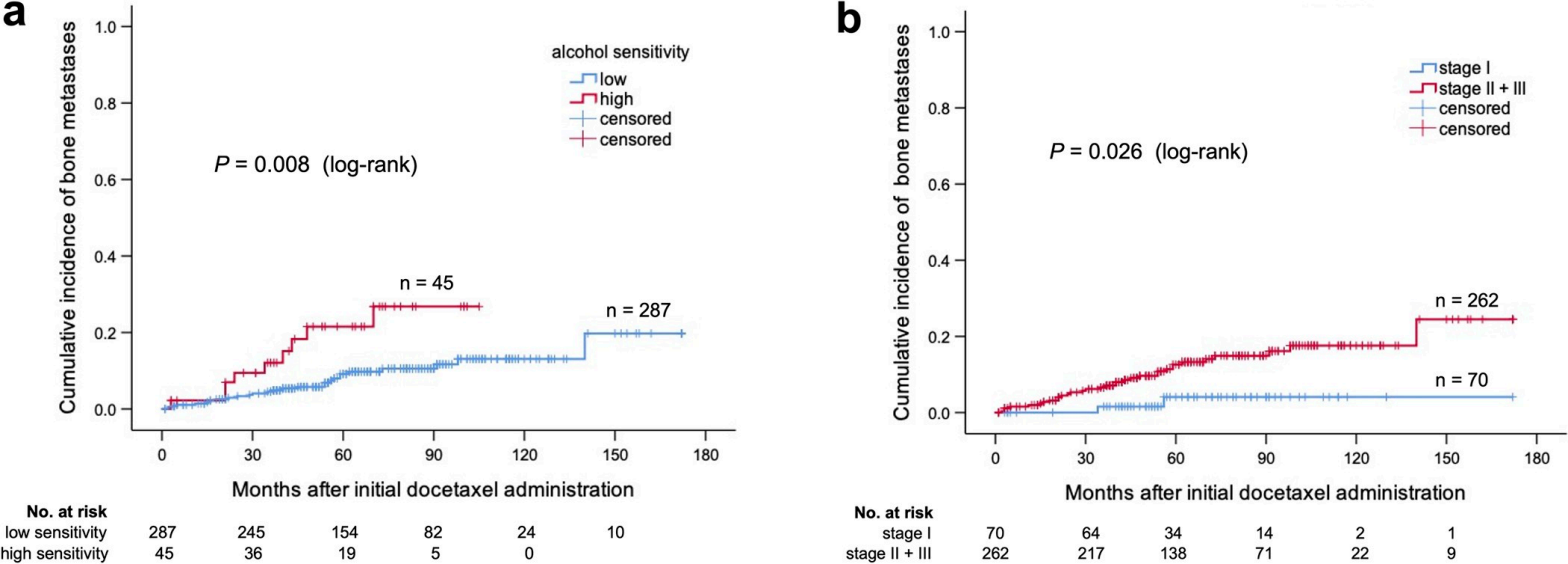
Cumulative incidence of bone metastases by alcohol sensitivity and clinical stages. (a) Cumulative incidence of bone metastases by alcohol sensitivity. (b) Cumulative incidence of bone metastases by clinical stage (stage I vs. stages II and III). The number below each figure is the number of patients at risk.

**Fig 3:**
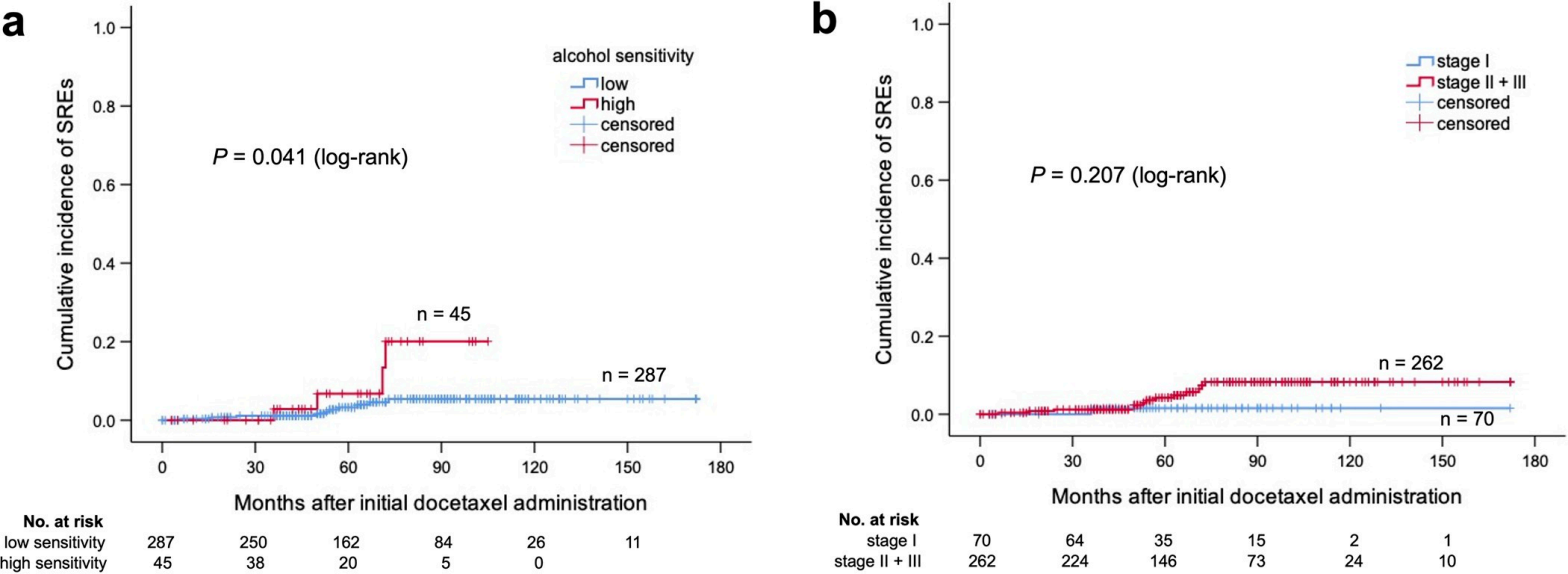
Cumulative incidence of SREs by alcohol sensitivity and clinical stage. (a) Cumulative incidence of SREs by alcohol sensitivity. (b) Cumulative incidence of SREs by clinical stage (stage I vs. stages II and III). The number below each figure is the number of patients at risk. SREs, skeletal-related events.

There were significant differences in the incidence of bone metastases between high and low sensitivities (*P* = 0.008) (Fig 2A) and between clinical stages (stage I vs. stages II and III) (*P* = 0.026) (Fig 2B). The high-sensitivity group had a significantly higher incidence of SREs (*P* = 0.041) (Fig 3A), whereas there was no significant difference in clinical stage (Fig 3B).

In univariate analysis, high clinical stage (stages II and III) and high sensitivity to alcohol were significant factors for bone metastases. In multivariate analysis, the hazard ratio (HR) of high clinical stage (stages II and III) for bone metastases was 4.449 (95% CI 1.066–18.564, *P* = 0.041). The HR of high sensitivity to alcohol for bone metastases was 2.721 (95% CI 1.268–5.841, *P* = 0.010). Both were significant prognostic factors for bone metastases (Table 6). There were no significant variables for SREs based on the Cox proportional hazards model in univariate analysis (Table 7).

**Table 6:** Univariate and multivariate analyses of risk factors for bone metastases.

Variables	Univariate analysis			Multivariate analysis		
	HR	95% CI	*P-*value*	HR	95% CI	*P-*value*
Age (< 50 vs. ≥ 50)	0.820	0.420–1.602	0.562			
cTNM stage (I vs. II and III)	4.409	1.057–18.393	0.042	4.449	1.066–18.564	0.041
ER (ER- vs. ER+)	1.429	0.624–3.271	0.399			
PgR (PgR- vs. PgR+)	0.996	0.502–1.977	0.991			
HER2 (HER2- vs. HER2+)	1.231	0.574–2.640	0.594			
Alcohol sensitivity (low vs. high)	2.698	1.256–5.793	0.011	2.721	1.268–5.841	0.010

ER, estrogen receptor; PgR, progesterone receptor; HER2, human epidermal growth factor receptor 2

*P-values were calculated using the Cox proportional hazards model.

**Table 7:** Univariate analyses of risk factors for SREs.

Variables	Univariate analysis		
	HR	95% CI	*P-*value*
Age (< 50 vs. ≥ 50)	0.462	0.160–1.332	0.153
cTNM stage (I vs. II and III)	3.423	0.448–26.174	0.236
ER (ER- vs. ER+)	2.014	0.451–9.004	0.359
PgR (PgR- vs. PgR+)	0.759	0.263–2.188	0.61
HER2 (HER2- vs. HER2+)	2.294	0.766–6.869	0.138
Alcohol sensitivity (low vs. high)	3.143	0.984–10.043	0.053

SREs, skeletal-related events; ER, estrogen receptor; PgR, progesterone receptor

HER2, human epidermal growth factor receptor 2

*P-values were calculated using the Cox proportional hazards model.

### SREs and MRONJ

**Fig 4:**
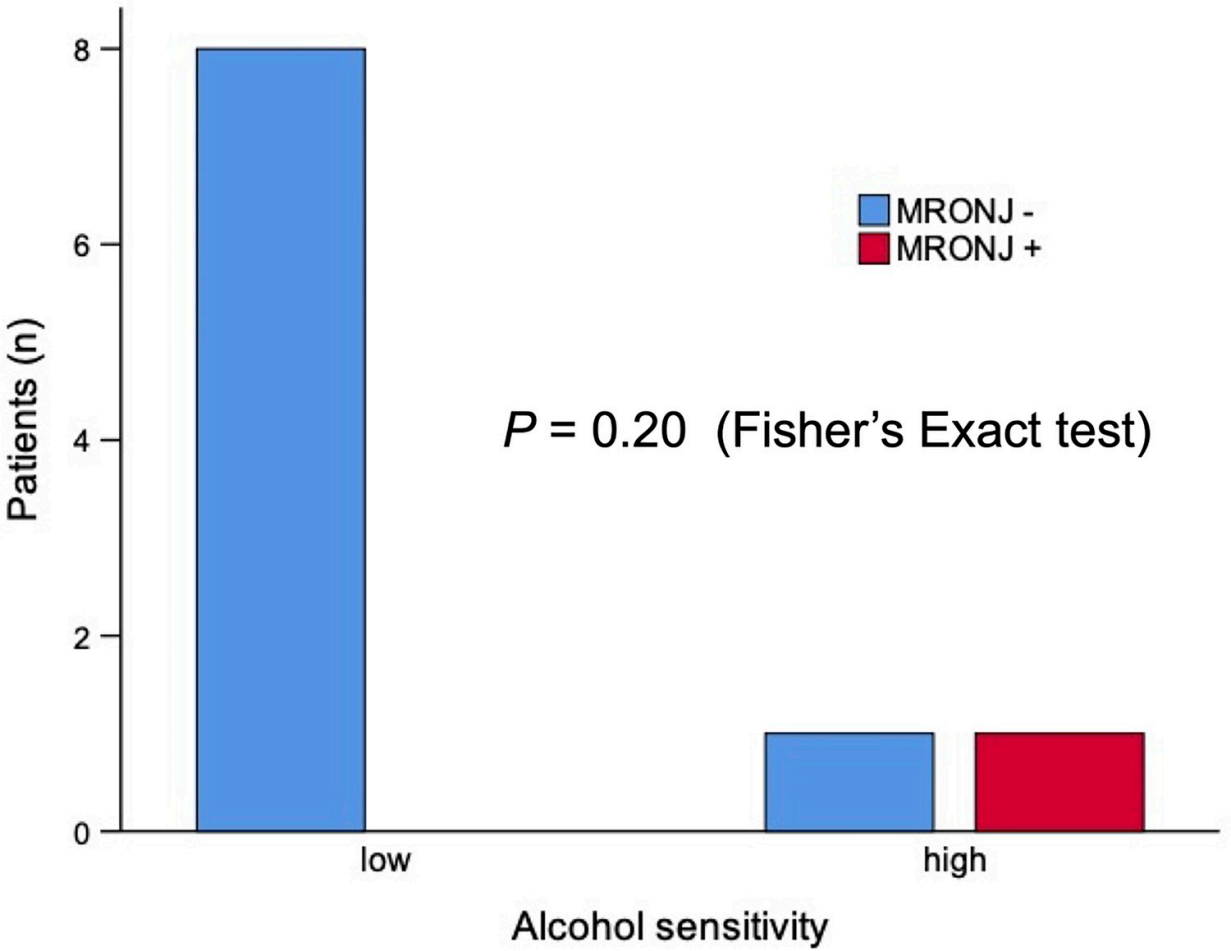
Alcohol-sensitivity and MRONJ in patients administered bone-modifying agents. MRONJ, medication-related osteonecrosis of the jaw.

No significant differences in the types of SREs were found by sensitivity. Pathological fracture was observed in one patient (primary staging: stage II) with high sensitivity to alcohol (Table 8). Bone-modifying agents, zoledronic acid or denosumab, were administered to 10 patients who developed bone metastases. MRONJ developed in one patient (primary staging: stage III) of the two (50%) patients with high sensitivity to alcohol, whereas no MRONJ was observed in eight patients with low sensitivity to alcohol. Fisher’s exact test demonstrated no significant differences between the two groups (*P* = 0.20) (Fig 4).

**Table 8:** Types of SREs and sensitivity to alcohol.

SREs	Low sensitivity (n = 26)	High sensitivity (n = 9)	*P*-value*
Pathological fracture	0 (0%)	1 (11.1%)	0.226
Pain or radiation	10 (38.5%)	3 (33.3%)	
None	16 (61.5%)	5 (55.6%)	

SREs, skeletal-related events

*P-value was calculated using the chi-square test.

## Discussion

Prognostic risk factors for bone metastases are a concern in breast cancer treatment worldwide [29–32]. Population-based studies in Denmark, the United Kingdom, and the United States revealed a greater incidence of bone metastases among patients with advanced stages at diagnosis of breast cancer [29, 31, 32]. A multicenter retrospective observational study in Japan reported that a large number of lymph node metastases and advanced disease stages or certain subtypes, such as luminal-HER2, HER2, and triple negative, are risk factors for bone metastases [30]. In the present study, advanced stage (stage I vs. stages II and III) was a significant prognostic factor for bone metastases, which is consistent with previous studies. In addition to stage, our multivariate analysis demonstrated that hypersensitivity to alcohol (low sensitivity vs. high sensitivity) was a significant prognostic factor for bone metastases in primary operable breast cancer patients (Table 6). Kaplan-Meier estimates stratified by alcohol sensitivity support the hypothesis that the potential influence of tumor stages on bone metastases is relatively weaker in high-sensitivity patients (Tables 2 and 3 and S1A and S1B Fig). Knowledge about sensitivity to alcohol may be useful for reducing the risk of MRONJ because earlier dental consultation and treatment are recommended for cancer patients even before developing bone metastases [8]. In the present study, no significant variables remained in the univariate analysis for SREs (Table 7). We compared types of SREs and development of MRONJ by sensitivity to alcohol. Pathological fracture and MRONJ were observed in patients with high sensitivity to alcohol, whereas no significant differences were found between the two groups (Table 8) (Fig 4). However, the numbers of patients with SREs or MRONJ were insufficient to obtain significant results because those with stage IV and recurrence were excluded from this study.

In Japan, the consumption of alcohol among women is less prevalent than among men [33, 34]. Patients with high sensitivity to alcohol may drink fewer alcoholic beverages because of the flush reaction. In addition to alcoholic beverages, diet can be a local source of acetaldehyde as some dairy products, fruits, vegetables, and alcohol-free beverages include acetaldehyde due to the production process or as a flavor compound [35, 36]. Furthermore, mouthwashes used as oral hygiene products often contain alcohol. A large meta-analysis reported that the frequent use of mouthwash was related to oral cancer (OR = 1.11) and oropharyngeal cancer (OR = 1.28), likely through the carcinogenic activity of acetaldehyde [37]. These routes for acetaldehyde exposure may affect patients with impaired ALDH2. Host metabolism and oral microbial metabolism also function in acetaldehyde production [35, 38]. As for acetaldehyde originating from oral microbiota, a previous study reported that professional oral care reduced the level of acetaldehyde in perioperative patients with esophageal cancer [39]. A recent population-based study indicated that rs671 polymorphism of *ALDH2* increased breast cancer risk independently even after adjusting for alcohol consumption [40]. Earlier dental consultation and treatment may be useful for patients with breast cancer. Furthermore, endogenous or environmental aldehydes were suggested to play a role in genome instability by inducing haploinsufficiency of *BRCA2*, a breast cancer susceptibility gene [41]. It was reported that those deficient in ALDH2 activity accumulate endogenous formaldehyde because mitochondrial ALDH2 is essential for the metabolic clearance of formaldehyde together with cytoplasmic alcohol dehydrogenase 5 in human hematopoiesis [42]. High sensitivity to alcohol may reflect more complex metabolic cascades related to impaired ALDH2 with genetic polymorphisms or specific environmental exposure.

The present study has several limitations. The assessment of hypersensitivity to alcohol was based on medical records of alcohol sensitivity rather than genotyping of the rs671 polymorphism of *ALDH2*, even though a flushing questionnaire had a sensitivity and specificity of approximately 90% for identifying ALDH2-deficient individuals among Japanese women [23, 24]. In the present study, the rate of patients with high sensitivity to alcohol was 14% (45/332). There are two possible explanations for our results: (a) patients with high sensitivity to alcohol had the inactive form of ALDH2 encoded by the rs671 polymorphism of *ALDH2*, and the other patients had the active form of ALDH2; or (b) patients with high sensitivity to alcohol had the inactive form of ALDH2, whereas a small number of ALDH2-deficient individuals were included among patients with low sensitivity to alcohol. Flushing after light drinking may disappear after a long history of drinking alcohol [18, 22]. Those with a long history of drinking may not have noticed their past flushing and received docetaxel with ethanol solution even though they have the inactive form of ALDH2. Thus, a lack of information about patients’ lifestyle, such as alcohol consumption, diet, and oral hygiene, is a limitation of this study. Another limitation is that mild cases of MRONJ may be underestimated. Although the risk of osteonecrosis of the jaw should be evaluated before starting bone-modifying agents and patients should receive regular oral care during this treatment [43], medical doctors may have consulted dentists only after being requested to do so by patients that developed oral problems. Finally, the number of patients was small because the study was conducted at a single hospital.

In conclusion, this study suggested that sensitivity to alcohol and clinical stages are significant prognostic factors for bone metastases of primary operable breast cancer. Further genetic investigations and metabolic analyses are necessary to clarify causality.

## Supporting information

S1 Fig Kaplan-Meier estimates of cumulative incidence of bone metastases after initial docetaxel administration, stratified by alcohol sensitivity (a, b) and by tumor stages (c, d, e). (a) low sensitivity: stage I vs. III (log-rank test, P = 0.003), stage II vs. III (P = 0.005), (b) high sensitivity: no significant differences between stages, (c) stage I (P = 0.101), (d) stage II (P = 0.017), (e) stage III (P = 0.125).
(PDF)

S2 Fig Kaplan-Meier estimates of cumulative incidence of SREs after initial docetaxel administration, stratified by alcohol sensitivity (a, b) and by tumor stages (c, d, e). (a) Low sensitivity: no significant differences between stages, (b) high sensitivity: no significant differences between stages, (c) stage I (P = 0.013), (d) stage II (P = 0.682), (e) stage III (P = 0.079).
(PDF)

S1 Dataset (XLSX)

## References

[1] Bray F, Ferlay J, Soerjomataram I, Siegel RL, Torre LA, Jemal A. Global cancer statistics 2018: GLOBOCAN estimates of incidence and mortality worldwide for 36 cancers in 185 countries. CA Cancer J Clin. 2018;68(6):394–424. Epub 2018/09/13. doi: 10.3322/caac.21492 . 30207593

[2] O’Carrigan B, Wong MH, Willson ML, Stockler MR, Pavlakis N, Goodwin A. Bisphosphonates and other bone agents for breast cancer. Cochrane Database Syst Rev. 2017;10:CD003474. Epub 2017/10/31. doi: 10.1002/14651858.CD003474.pub4 ; PubMed Central PMCID: PMC6485886. 29082518 PMC6485886

[3] Macedo F, Ladeira K, Pinho F, Saraiva N, Bonito N, Pinto L, et al. Bone Metastases: An Overview. Oncol Rev. 2017;11(1):321. Epub 2017/06/07. doi: 10.4081/oncol.2017.321 ; PubMed Central PMCID: PMC5444408. 28584570 PMC5444408

[4] Kajizono M, Sada H, Sugiura Y, Soga Y, Kitamura Y, Matsuoka J, et al. Incidence and Risk Factors of Osteonecrosis of the Jaw in Advanced Cancer Patients after Treatment with Zoledronic Acid or Denosumab: A Retrospective Cohort Study. Biol Pharm Bull. 2015;38(12):1850–5. Epub 2015/12/04. doi: 10.1248/bpb.b15-00385 . 26632176

[5] Khan AA, Morrison A, Hanley DA, Felsenberg D, McCauley LK, O’Ryan F, et al. Diagnosis and management of osteonecrosis of the jaw: a systematic review and international consensus. J Bone Miner Res. 2015;30(1):3–23. Epub 2014/11/22. doi: 10.1002/jbmr.2405 . 25414052

[6] Yarom N, Shapiro CL, Peterson DE, Van Poznak CH, Bohlke K, Ruggiero SL, et al. Medication-Related Osteonecrosis of the Jaw: MASCC/ISOO/ASCO Clinical Practice Guideline. J Clin Oncol. 2019;37(25):2270–90. Epub 2019/07/23. doi: 10.1200/JCO.19.01186 . 31329513

[7] Ruggiero SL, Dodson TB, Fantasia J, Goodday R, Aghaloo T, Mehrotra B, et al. American Association of Oral and Maxillofacial Surgeons position paper on medication-related osteonecrosis of the jaw—2014 update. J Oral Maxillofac Surg. 2014;72(10):1938–56. Epub 2014/09/23. doi: 10.1016/j.joms.2014.04.031 . 25234529

[8] Ikesue H, Mouri M, Tomita H, Hirabatake M, Ikemura M, Muroi N, et al. Associated characteristics and treatment outcomes of medication-related osteonecrosis of the jaw in patients receiving denosumab or zoledronic acid for bone metastases. Support Care Cancer. 2021;29(8):4763–72. Epub 2021/02/03. doi: 10.1007/s00520-021-06018-x ; PubMed Central PMCID: PMC8236436. 33527228 PMC8236436

[9] Owosho AA, Liang STY, Sax AZ, Wu K, Yom SK, Huryn JM, et al. Medication-related osteonecrosis of the jaw: An update on the memorial sloan kettering cancer center experience and the role of premedication dental evaluation in prevention. Oral Surg Oral Med Oral Pathol Oral Radiol. 2018;125(5):440–5. Epub 2018/03/28. doi: 10.1016/j.oooo.2018.02.003 ; PubMed Central PMCID: PMC7518027. 29580668 PMC7518027

[10] Urade M, Tanaka N, Furusawa K, Shimada J, Shibata T, Kirita T, et al. Nationwide survey for bisphosphonate-related osteonecrosis of the jaws in Japan. J Oral Maxillofac Surg. 2011;69(11):e364–71. Epub 2011/07/26. doi: 10.1016/j.joms.2011.03.051 . 21782307

[11] Drudge-Coates L, Van den Wyngaert T, Schiodt M, van Muilekom HAM, Demonty G, Otto S. Preventing, identifying, and managing medication-related osteonecrosis of the jaw: a practical guide for nurses and other allied healthcare professionals. Support Care Cancer. 2020;28(9):4019–29. Epub 2020/04/21. doi: 10.1007/s00520-020-05440-x ; PubMed Central PMCID: PMC7378104. 32307659 PMC7378104

[12] Shemanko CS, Cong Y, Forsyth A. What Is Breast in the Bone? International journal of molecular sciences. 2016;17(10). Epub 2016/10/27. doi: 10.3390/ijms17101764 ; PubMed Central PMCID: PMC5085788. 27782069 PMC5085788

[13] Chen YC, Sosnoski DM, Mastro AM. Breast cancer metastasis to the bone: mechanisms of bone loss. Breast Cancer Res. 2010;12(6):215. Epub 2010/12/24. doi: 10.1186/bcr2781 ; PubMed Central PMCID: PMC3046443. 21176175 PMC3046443

[14] Yamaguchi J, Hasegawa Y, Kawasaki M, Masui T, Kanoh T, Ishiguro N, et al. ALDH2 polymorphisms and bone mineral density in an elderly Japanese population. Osteoporos Int. 2006;17(6):908–13. Epub 2006/03/08. doi: 10.1007/s00198-006-0077-2 . 16520888

[15] Shimizu Y, Sakai A, Menuki K, Mori T, Isse T, Oyama T, et al. Reduced bone formation in alcohol-induced osteopenia is associated with elevated p21 expression in bone marrow cells in aldehyde dehydrogenase 2-disrupted mice. Bone. 2011;48(5):1075–86. Epub 2011/01/25. doi: 10.1016/j.bone.2011.01.008 . 21256255

[16] Hoshi H, Hao W, Fujita Y, Funayama A, Miyauchi Y, Hashimoto K, et al. Aldehyde-stress resulting from Aldh2 mutation promotes osteoporosis due to impaired osteoblastogenesis. J Bone Miner Res. 2012;27(9):2015–23. Epub 2012/04/18. doi: 10.1002/jbmr.1634 . 22508505

[17] Chen CH, Ferreira JC, Gross ER, Mochly-Rosen D. Targeting aldehyde dehydrogenase 2: new therapeutic opportunities. Physiol Rev. 2014;94(1):1–34. Epub 2014/01/03. doi: 10.1152/physrev.00017.2013 ; PubMed Central PMCID: PMC3929114. 24382882 PMC3929114

[18] Brooks PJ, Enoch MA, Goldman D, Li TK, Yokoyama A. The alcohol flushing response: an unrecognized risk factor for esophageal cancer from alcohol consumption. PLoS Med. 2009;6(3):e50. Epub 2009/03/27. doi: 10.1371/journal.pmed.1000050 ; PubMed Central PMCID: PMC2659709. 19320537 PMC2659709

[19] Harada S, Agarwal DP, Goedde HW. Aldehyde dehydrogenase deficiency as cause of facial flushing reaction to alcohol in Japanese. Lancet. 1981;2(8253):982. Epub 1981/10/31. doi: 10.1016/s0140-6736(81)91172-7 . 6117742

[20] Oota H, Pakstis AJ, Bonne-Tamir B, Goldman D, Grigorenko E, Kajuna SL, et al. The evolution and population genetics of the ALDH2 locus: random genetic drift, selection, and low levels of recombination. Ann Hum Genet. 2004;68(Pt 2):93–109. Epub 2004/03/11. doi: 10.1046/j.1529-8817.2003.00060.x . 15008789

[21] Peng GS, Chen YC, Wang MF, Lai CL, Yin SJ. ALDH2*2 but not ADH1B*2 is a causative variant gene allele for Asian alcohol flushing after a low-dose challenge: correlation of the pharmacokinetic and pharmacodynamic findings. Pharmacogenet Genomics. 2014;24(12):607–17. Epub 2014/11/05. doi: 10.1097/FPC.0000000000000096 . 25365528

[22] Yokoyama T, Yokoyama A, Kato H, Tsujinaka T, Muto M, Omori T, et al. Alcohol flushing, alcohol and aldehyde dehydrogenase genotypes, and risk for esophageal squamous cell carcinoma in Japanese men. Cancer Epidemiol Biomarkers Prev. 2003;12(11 Pt 1):1227–33. Epub 2003/12/04. . 14652286

[23] Yokoyama A, Kato H, Yokoyama T, Igaki H, Tsujinaka T, Muto M, et al. Esophageal squamous cell carcinoma and aldehyde dehydrogenase-2 genotypes in Japanese females. Alcoholism, clinical and experimental research. 2006;30(3):491–500. Epub 2006/02/28. doi: 10.1111/j.1530-0277.2006.00053.x . 16499490

[24] Yokoyama A, Omori T, Yokoyama T. Alcohol and aldehyde dehydrogenase polymorphisms and a new strategy for prevention and screening for cancer in the upper aerodigestive tract in East Asians. Keio J Med. 2010;59(4):115–30. Epub 2010/12/29. doi: 10.2302/kjm.59.115 . 21187698

[25] Ogawa C, Yatabe M, Inoue M, Hirose S, Ohashi Y, Yachi Y, et al. [Comparison of Chemical Behavior of Original and Generic Docetaxel Formulations as Non-alcoholic Preparations: Discussion about Diluent Solvents for Docetaxel]. Yakugaku Zasshi. 2018;138(7):973–84. Epub 2018/07/03. doi: 10.1248/yakushi.18-00006 . 29962477

[26] Yagi T, Fujiishi K, Hasegawa A, Otsuka T, Yoshinami T, Nishio M, et al. Aldehyde dehydrogenase 2 genotype in tolerability of alcohol contained in paclitaxel in Japanese breast cancer patients. Breast Cancer. 2019;26(2):229–34. Epub 2018/10/24. doi: 10.1007/s12282-018-0918-9 . 30350259

[27] Takeshima K, Nishiwaki Y, Suda Y, Niki Y, Sato Y, Kobayashi T, et al. A missense single nucleotide polymorphism in the ALDH2 gene, rs671, is associated with hip fracture. Sci Rep. 2017;7(1):428. Epub 2017/03/30. doi: 10.1038/s41598-017-00503-2 ; PubMed Central PMCID: PMC5428735. 28348376 PMC5428735

[28] Coleman RE, McCloskey EV. Bisphosphonates in oncology. Bone. 2011;49(1):71–6. Epub 2011/02/16. doi: 10.1016/j.bone.2011.02.003 . 21320652

[29] Hernandez RK, Wade SW, Reich A, Pirolli M, Liede A, Lyman GH. Incidence of bone metastases in patients with solid tumors: analysis of oncology electronic medical records in the United States. BMC Cancer. 2018;18(1):44. Epub 2018/01/08. doi: 10.1186/s12885-017-3922-0 ; PubMed Central PMCID: PMC5756362. 29306325 PMC5756362

[30] Yamashiro H, Takada M, Nakatani E, Imai S, Yamauchi A, Tsuyuki S, et al. Prevalence and risk factors of bone metastasis and skeletal related events in patients with primary breast cancer in Japan. Int J Clin Oncol. 2014;19(5):852–62. Epub 2013/12/03. doi: 10.1007/s10147-013-0643-5 . 24292334

[31] Hagberg KW, Taylor A, Hernandez RK, Jick S. Incidence of bone metastases in breast cancer patients in the United Kingdom: results of a multi-database linkage study using the general practice research database. Cancer Epidemiol. 2013;37(3):240–6. Epub 2013/02/19. doi: 10.1016/j.canep.2013.01.006 . 23416031

[32] Jensen AO, Jacobsen JB, Norgaard M, Yong M, Fryzek JP, Sorensen HT. Incidence of bone metastases and skeletal-related events in breast cancer patients: a population-based cohort study in Denmark. BMC Cancer. 2011;11:29. Epub 2011/01/26. doi: 10.1186/1471-2407-11-29 ; PubMed Central PMCID: PMC3037922. 21261987 PMC3037922

[33] Osaki Y, Kinjo A, Higuchi S, Matsumoto H, Yuzuriha T, Horie Y, et al. Prevalence and Trends in Alcohol Dependence and Alcohol Use Disorders in Japanese Adults; Results from Periodical Nationwide Surveys. Alcohol Alcohol. 2016;51(4):465–73. Epub 2016/02/14. doi: 10.1093/alcalc/agw002 . 26873982

[34] Yokoyama A, Yokoyama T, Omori T, Maesato H, Takimura T, Iwahara C, et al. Endoscopic screening using esophageal iodine staining and genotypes of ADH1B and ALDH2 in Japanese alcohol-dependent women. PLoS One. 2019;14(1):e0210546. Epub 2019/01/11. doi: 10.1371/journal.pone.0210546 ; PubMed Central PMCID: PMC6328133. 30629674 PMC6328133

[35] Nieminen MT, Salaspuro M. Local Acetaldehyde-An Essential Role in Alcohol-Related Upper Gastrointestinal Tract Carcinogenesis. Cancers (Basel). 2018;10(1). Epub 2018/01/06. doi: 10.3390/cancers10010011 ; PubMed Central PMCID: PMC5789361. 29303995 PMC5789361

[36] Uebelacker M, Lachenmeier DW. Quantitative determination of acetaldehyde in foods using automated digestion with simulated gastric fluid followed by headspace gas chromatography. J Autom Methods Manag Chem. 2011;2011:907317. Epub 2011/07/13. doi: 10.1155/2011/907317 ; PubMed Central PMCID: PMC3124883. 21747735 PMC3124883

[37] Boffetta P, Hayes RB, Sartori S, Lee YC, Muscat J, Olshan A, et al. Mouthwash use and cancer of the head and neck: a pooled analysis from the International Head and Neck Cancer Epidemiology Consortium. Eur J Cancer Prev. 2016;25(4):344–8. Epub 2015/08/15. doi: 10.1097/CEJ.0000000000000179 ; PubMed Central PMCID: PMC4752930. 26275006 PMC4752930

[38] Tagaino R, Washio J, Abiko Y, Tanda N, Sasaki K, Takahashi N. Metabolic property of acetaldehyde production from ethanol and glucose by oral Streptococcus and Neisseria. Sci Rep. 2019;9(1):10446. Epub 2019/07/20. doi: 10.1038/s41598-019-46790-9 ; PubMed Central PMCID: PMC6639336. 31320675 PMC6639336

[39] Tanda N, Washio J, Kamei T, Akazawa K, Takahashi N, Koseki T. Professional Oral Care Reduces Carcinogenic Acetaldehyde Levels in Mouth Air of Perioperative Esophageal Cancer Patients: A Prospective Comparative Study. The Tohoku journal of experimental medicine. 2019;249(1):75–83. Epub 2019/10/01. doi: 10.1620/tjem.249.75 . 31564686

[40] Park B, Kim JH, Lee ES, Jung SY, Lee SY, Kang HS, et al. Role of aldehyde dehydrogenases, alcohol dehydrogenase 1B genotype, alcohol consumption, and their combination in breast cancer in East-Asian women. Sci Rep. 2020;10(1):6564. Epub 2020/04/18. doi: 10.1038/s41598-020-62361-9 ; PubMed Central PMCID: PMC7162854. 32300124 PMC7162854

[41] Tan SLW, Chadha S, Liu Y, Gabasova E, Perera D, Ahmed K, et al. A Class of Environmental and Endogenous Toxins Induces BRCA2 Haploinsufficiency and Genome Instability. Cell. 2017;169(6):1105–18 e15. Epub 2017/06/03. doi: 10.1016/j.cell.2017.05.010 ; PubMed Central PMCID: PMC5457488. 28575672 PMC5457488

[42] Dingler FA, Wang M, Mu A, Millington CL, Oberbeck N, Watcham S, et al. Two Aldehyde Clearance Systems Are Essential to Prevent Lethal Formaldehyde Accumulation in Mice and Humans. Mol Cell. 2020;80(6):996–1012 e9. Epub 2020/11/05. doi: 10.1016/j.molcel.2020.10.012 ; PubMed Central PMCID: PMC7758861. 33147438 PMC7758861

[43] Shimoi T, Nagai SE, Yoshinami T, Takahashi M, Arioka H, Ishihara M, et al. The Japanese Breast Cancer Society Clinical Practice Guidelines for systemic treatment of breast cancer, 2018 edition. Breast Cancer. 2020;27(3):322–31. Epub 2020/04/03. doi: 10.1007/s12282-020-01085-0 ; PubMed Central PMCID: PMC8062371. 32240526 PMC8062371

