# Supplementary figures and images for: Influence of alcohol sensitivity on bone metastases and skeletal-related events in primary operable breast cancer: A retrospective cohort study
Source: PLoS One. 2022 Jun 3;17(6):e0269335. doi: 10.1371/journal.pone.0269335 (PMC9165843; doi:10.1371/journal.pone.0269335)

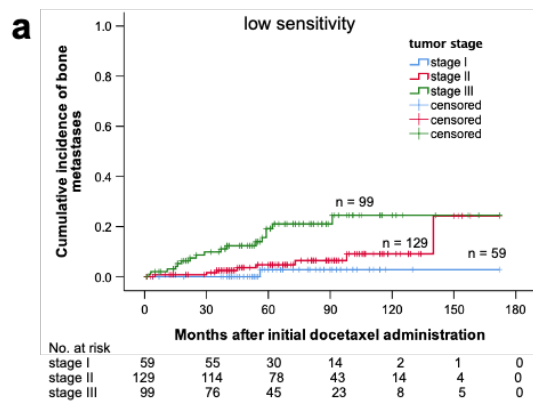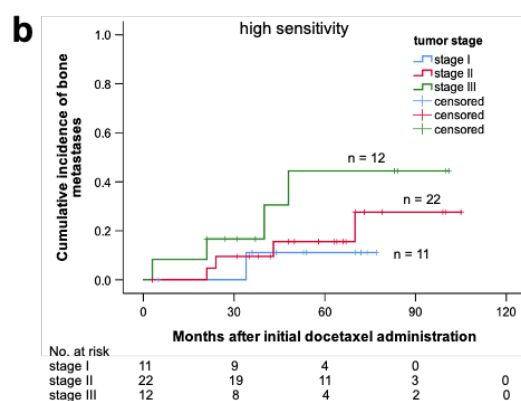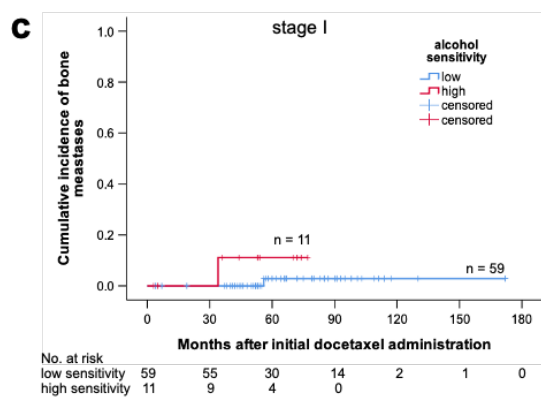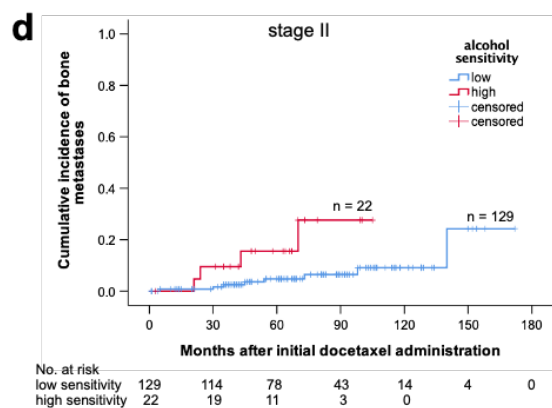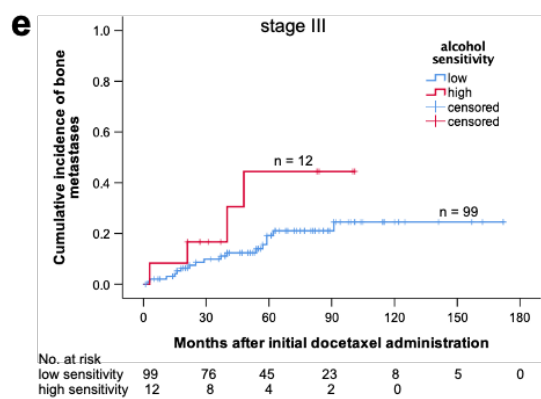

Supplement: S1 Fig — Kaplan-Meier estimates of cumulative incidence of bone metastases after initial docetaxel administration, stratified by alcohol sensitivity (a, b) and by tumor stages (c, d, e). (a) low sensitivity: stage I vs. III (log-rank test, P = 0.003), stage II vs. III (P = 0.005), (b) high sensitivity: no significant differences between stages, (c) stage I (P = 0.101), (d) stage II (P = 0.017), (e) stage III (P = 0.125). (PDF) [file pone.0269335.s001.pdf]

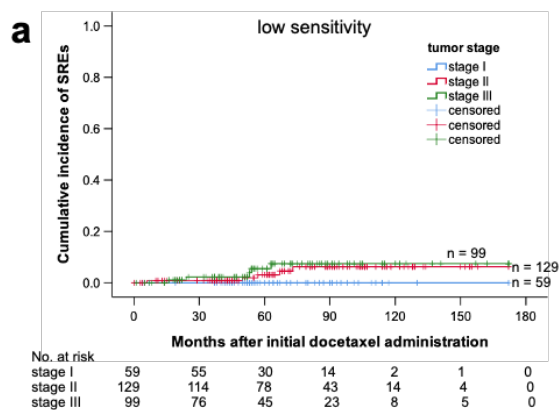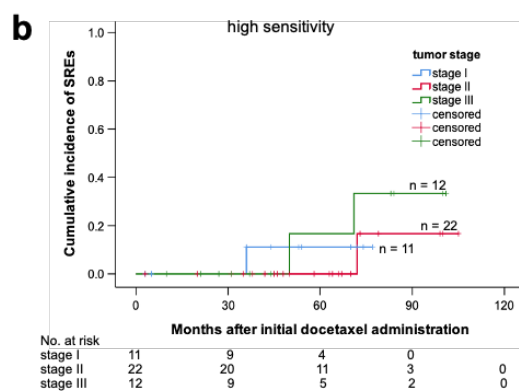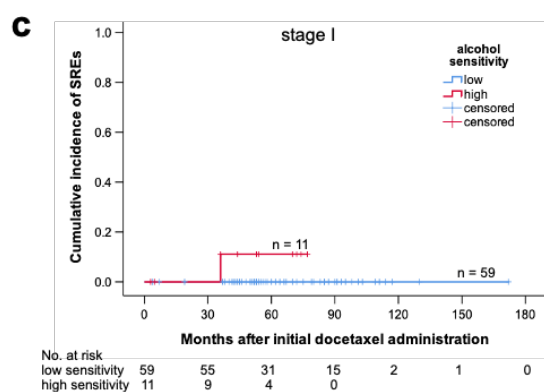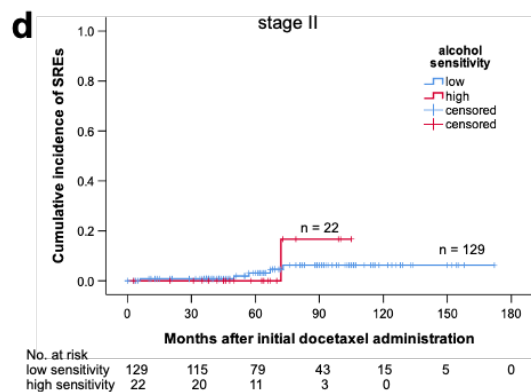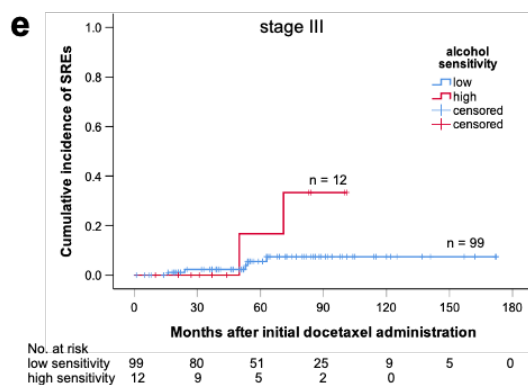

Supplement: S2 Fig — Kaplan-Meier estimates of cumulative incidence of SREs after initial docetaxel administration, stratified by alcohol sensitivity (a, b) and by tumor stages (c, d, e). (a) Low sensitivity: no significant differences between stages, (b) high sensitivity: no significant differences between stages, (c) stage I (P = 0.013), (d) stage II (P = 0.682), (e) stage III (P = 0.079). (PDF) [file pone.0269335.s002.pdf]
